# Ensuring equitable access to COVID-19 vaccines

**DOI:** 10.2471/BLT.20.021220

**Published:** 2020-12-01

## Abstract

Significant challenges need to be overcome to ensure eventual COVID-19 vaccines get to everyone who needs them. Andréia Azevedo Soares reports.

For Dr Simone Santana, a vaccine against the novel coronavirus disease 2019 (COVID-19) cannot come soon enough.

Working as a dentist at the Cecília Donnangelo medical centre in Vargem Grande, a neighbourhood in Rio de Janeiro, Brazil, the country with the third highest number of reported COVID-19 infections in the world, Santana feels exposed each time a patient comes in.

“I wear personal protective equipment and avoid using the drill as much as I can or anything else that can spread aerosols around the room, but there are moments when I’m afraid,” she says.

Santana is hoping that one or more of the clinical trials of COVID-19 vaccine candidates currently being conducted will result in a safe and effective vaccine that can then be distributed to all those who need it.

Such an outcome would represent a break with historical precedent.

“Experiences from past pandemics show that low- and middle-income countries tend to wait longer than their wealthier counterparts to get access to vaccines and other vital health products,” says Claudia Nannei, an expert in health programme management at the World Health Organization (WHO).

In some cases, delays have been exacerbated by higher-income countries pre-emptively buying up vaccine supplies. This was the case, for example, during the 2009 H1N1 pandemic when high-income countries placed large advance orders for 2009-H1N1 vaccine, eventually acquiring the bulk of the vaccine manufactured.

To prevent low- and middle-income countries being left behind in the current pandemic, governments, international organizations, philanthropists, civil society and other stakeholders have come together in an initiative designed to ensure the research, development and manufacture of a wide range of COVID-19 vaccine candidates, negotiate their pricing and – should any be approved for use – ensure their equitable distribution.

Referred to simply as COVAX, the initiative is one of three pillars of the Access to COVID-19 Tools (ACT) Accelerator, a global collaboration aimed at accelerating the development, production and equitable access to COVID-19 tests, treatments and vaccines.

The basic idea behind COVAX, which is led by the Coalition for Epidemic Preparedness Innovations (CEPI), Gavi, the Vaccine Alliance, and WHO and supported by the Bill & Melinda Gates Foundation, the Foundation for Innovative New Diagnostics, the Global Fund, Unitaid, Wellcome and the World Bank, is to bring countries and economies together to pool resources, support the development and manufacture of candidate vaccines and ensure their equitable distribution to countries that might otherwise be left behind.

“We’ve had a level of support that’s incredible.”

Aurélia Nguyen

As of early November, 186 countries and economies, 94 of which declare self-financing and 92 requiring funding support, had joined COVAX’s vaccine procurement and delivery mechanism, known as the COVAX Facility.

“We’ve had a level of support that’s incredible,” says Aurélia Nguyen, managing director of the Office of the COVAX Facility at Gavi, pointing out that most of the world’s global population is represented.

The initial aim of COVAX is to make 2 billion doses of vaccine available by the end of 2021, 1 billion of which would be distributed to people in low-income countries. The hope is that this level of distribution will be sufficient to protect those at highest risk of serious illness or death. As more vaccine is manufactured, more will be distributed in 2022.

To achieve its aims, COVAX will have to overcome significant challenges, the first being the development and regulatory approval of vaccines that are safe and effective.

It has been estimated that around 1 in 10 vaccines make it from the laboratory to final approval. In the hope of overcoming those odds, CEPI – which is responsible for vaccine development and manufacture within COVAX – is spreading its development bets across nine candidate vaccines based on four distinct bio-technological platforms.

Assuming a successful candidate or candidates emerge, the next challenge will be making enough vaccine to meet demand. “We want to establish the biggest global manufacturing footprint possible,” says Richard Wilder, General Counsel and Head of Business Development at CEPI, which is currently working to identify manufacturers worldwide, including those in low- and middle-income countries.

Global manufacturing will not only ensure large-volume production; it will also reduce the possibility that some countries hoard the vaccines they produce. “In the past, countries manufacturing vaccines, have decided to prevent their export,” says Wilder, explaining that CEPI intentionally chose vaccines that were being developed in different locations around the globe to prevent this happening.

In order to meet the anticipated demand, many manufacturers have already started production of vaccines that may never be approved for sale – so-called manufacturing at risk.

To encourage such manufacturing, and to ensure that the vaccine produced is priced affordably for the 92 countries requiring funding support, Gavi set up a financing mechanism called the Gavi Advance Market Commitment for COVID-19 Vaccines. The mechanism provides donor-funded commitments to purchase specific candidate vaccines before they are licensed.

COVAX Facility participants are also hoping to keep prices down through pooled procurement – the governments are clubbing together to exert their combined purchasing power. However, pooled procurement will not work as well if sellers have options for transacting business bilaterally.

Some countries are pursuing bilateral deals to secure vaccine for their own populations, the United States of America (USA), which has so far not joined the COVAX Facility, being a notable example.

According to WHO estimates, countries have pre-ordered around 2 billion doses of different vaccine candidates through bilateral deals. Such demand risks putting upward pressure on prices while limiting opportunities for the COVAX Facility to secure vaccine supply.

Most of the bilateral deals are being transacted behind closed doors, but some indication of how high prices may go is offered by the deal announced in July between the government of the USA and the pharmaceutical company Pfizer and biotech partner BioNTech SE for their candidate vaccine. The government placed an initial order for 100 million doses at a cost of US$ 1.95 billion, enough to immunize 50 million people.

“We want to establish the biggest global manufacturing footprint possible.”

Richard Wilder

Once purchased, vaccines will need to be distributed. Here too, challenges are expected, particularly for vaccines that require special handling. For example, some vaccines based on messenger ribonucleic acid (mRNA) technology require storage at −70°C.

According to Nguyen, emerging data suggest that most COVID-19 vaccines should be able to operate in a −20°C or 2-8°C cold chain for at least a few months. “They could be shipped at those levels, then distributed and administered within countries using existing infrastructure,” she says.

Facility participants being supported via the Gavi COVAX Advance Market Commitment will have access to vaccine refrigerators that can operate on electricity grids or solar energy, 43 000 of which have been installed by Gavi over the past four years; another 5000 are scheduled for installation by the end of 2020.

Finally, countries will be faced with the challenges of effectively promoting and delivering vaccine and monitoring safety and effectiveness. According to Katherine O’Brien, director of the immunization, vaccines and biologicals department at WHO, the Organization has been supporting preparatory work for country delivery, and decision-making around vaccination as well as publishing guidance and frameworks.

“Challenges will vary from country to country, but certain considerations are likely to be common to all,” O’Brien says.

For example, because there will not be enough vaccine to meet all demands at once (the initial plan is to provide COVAX Facility participants with enough vaccine to cover 20% of their populations), certain groups will have to be prioritized.

WHO’s Strategic Advisory Group of Experts (SAGE) on Immunization recommends making health workers at high risk of infection the highest priority, followed by elderly people (the exact age will vary by country) with the highest risk of serious disease or death and then adults with underlying conditions.

“The SAGE recommendations are based on a thorough review of evidence and on the values framework released by WHO in the summer,” O’Brien says. “This provides countries with a solid foundation for policy development which will be critical for ensuring the public’s confidence in these decisions.”

Ensuring information accuracy and transparency – including transparency about likely efficacy – while at the same time countering misinformation, will also be vital to establishing public trust. Finally, post-marketing surveillance to monitor for adverse events will be crucial, especially given the ground-breaking nature of the technologies being used.

As daunting as the different challenges outlined may be, the prospects of overcoming them are likely to improve if the global public health community comes together in a spirit of solidarity. COVAX and the ACT Accelerator, of which it is part, are compelling examples of how such solidarity can be leveraged.

It was in a spirit of solidarity that Simone Santana signed up for the trial of a candidate vaccine being developed by AstraZeneca and Oxford University in June.

“I’m aware of the risks, but we have to beat this virus if we want to get back to normal living,” she says.

Brazil joined the COVAX Facility in September.

**Figure:**
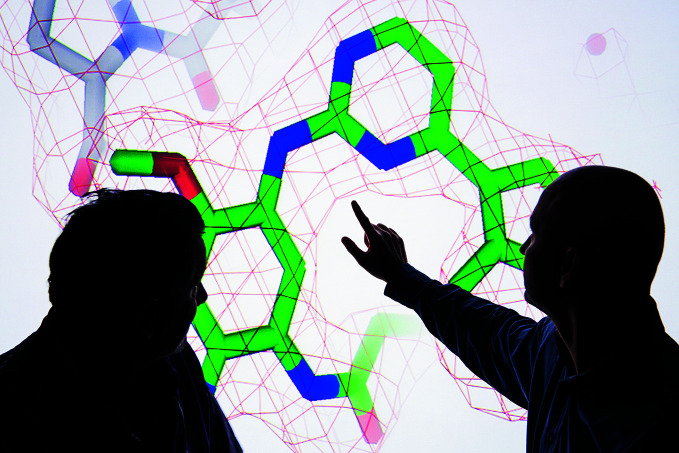
Scientists discuss one of the COVAX candidate compounds.

Gavi / Marco Betti

**Figure:**
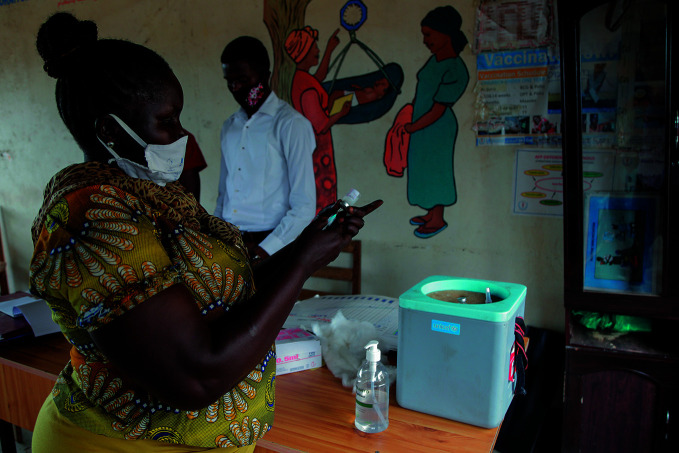
A nurse in Juba, South Sudan, collects vaccines from a cold box.

UNICEF / Charles Obel

